# Optimizing Vancomycin Dosing in Continuous Renal Replacement Therapy: A Systematic Review of Population Pharmacokinetic Studies in Adult Critically Ill Patients

**DOI:** 10.3390/pharmaceutics18030322

**Published:** 2026-03-03

**Authors:** Nursel Sürmelioğlu, Sevgin Memili, Karel Allegaert, Nadir Yalçın

**Affiliations:** 1 Department of Clinical Pharmacy, Faculty of Pharmacy, Çukurova University, Adana 01330, Türkiye; sevginmemili@gmail.com; 2 Department of Clinical Pharmacy, Faculty of Pharmacy, Hacettepe University, Ankara 06100, Türkiye; 3 Department of Pharmaceutical and Pharmacological Sciences, KU Leuven, 3000 Leuven, Belgium; karel.allegaert@kuleuven.be; 4 Department of Development and Regeneration, KU Leuven, 3000 Leuven, Belgium; 5 Child and Youth Institute, KU Leuven, 3000 Leuven, Belgium; 6 Department of Clinical Pharmacy, Erasmus MC, 3000 Rotterdam, The Netherlands

**Keywords:** continuous renal replacement therapy, population pharmacokinetics, model-informed precision dosing, adult, vancomycin

## Abstract

**Background:** Vancomycin dosing during continuous renal replacement therapy (CRRT) remains challenging due to profound pharmacokinetic (PK) variability and lack of standardized guidance. Population pharmacokinetic (PopPK) models provide a quantitative framework to identify covariates affecting drug disposition and support individualized dosing. **Methods:** This systematic review, registered in PROSPERO (CRD420250655157), comprehensively identified PopPK studies evaluating vancomycin in critically ill adults undergoing CRRT. PubMed, Embase, Web of Science, and Cochrane Library were searched from inception to 28 February 2025. Eligible studies reported PopPK analyses or simulations providing PK parameters and/or dosing recommendations. Data were extracted on study characteristics, CRRT settings, PK findings, and dose optimization strategies. In addition, covariates were categorized based on whether they were merely explored or statistically confirmed within the respective PopPK models, as commonly reported in similar pharmacometrics studies. Due to the methodological nature of population pharmacokinetic model development studies, no standardized risk-of-bias tool was applied; instead, a structured descriptive methodological appraisal was performed. Results were synthesized narratively given the heterogeneity in structural models, covariate strategies, and CRRT modalities. **Results:** Twelve PopPK studies published between 2013 and 2023 met the inclusion criteria. Considerable heterogeneity was observed across study designs, CRRT modalities, and dosing strategies. Reported vancomycin clearance ranged from 0.7 to 3.0 L/h, and volume of distribution from 0.8 L/kg to >100 L. Effluent rate consistently emerged as the primary determinant of clearance, while residual diuresis, albumin concentration, and vasopressor use acted as relevant covariates. Loading doses of 25–30 mg/kg (up to 35 mg/kg at high effluent rates) and effluent-adjusted maintenance regimens achieved therapeutic AUC24/MIC targets more consistently when supported by early and repeated therapeutic drug monitoring (TDM). **Conclusions:** Vancomycin PK during CRRT is highly variable and driven by effluent intensity and patient-specific factors. Fixed regimens are inadequate; individualized dosing guided by effluent flow, renal function, and TDM is essential for optimal exposure. Prospective, multicenter PopPK studies integrating pharmacodynamic targets and clinical outcomes are warranted to refine and validate CRRT-specific dosing strategies.

## 1. Introduction

Vancomycin is widely used as a first-line therapy for serious infections caused by Gram-positive pathogens and is an essential component of broad-spectrum antibiotic regimens in septic patients [1,2]. The drug is primarily eliminated through glomerular filtration, and excessive exposure has been associated with an increased risk of acute kidney injury [3]. In contemporary clinical practice, AUC-guided monitoring (target AUC/MIC 400–600) has largely replaced trough-based approaches, reflecting a shift toward exposure-driven dosing optimization in adult patients. Sepsis and septic shock may significantly alter the pharmacokinetics (PK) of hydrophilic agents such as vancomycin due to changes in volume of distribution and renal clearance [4]. Moreover, the frequent occurrence of acute kidney injury (AKI) in this setting necessitates the use of continuous renal replacement therapy (CRRT). Given that vancomycin is predominantly renally cleared, CRRT substantially affects its clearance [2]. Altered pharmacokinetics during CRRT may lead not only to subtherapeutic vancomycin exposure—particularly against resistant pathogens such as MRSA—but also to supratherapeutic concentrations in some patients, reflecting the high interindividual variability observed in this setting. Despite increasing clinical use of CRRT, antimicrobial dosing remains inconsistent, largely due to variability in patient characteristics, CRRT modalities, and the lack of standardized dosing guidance [1,5,6].

A recent mixed-methods study highlighted these gaps, demonstrating wide heterogeneity in clinician knowledge, limited utilization of therapeutic drug monitoring (TDM), and an urgent need for context-specific dosing frameworks for antibiotics during CRRT [7]. Existing literature has documented marked interpatient variability in vancomycin concentrations during CRRT, yet data describing vancomycin PK in this population remain limited [5]. Understanding the impact of CRRT on vancomycin PK is essential for optimizing dosing regimens [8]. To address this knowledge gap, population pharmacokinetic (PopPK) studies are needed to characterize vancomycin disposition during different CRRT modalities and to provide the structural framework for model-informed precision dosing (MIPD). When combined with Bayesian estimation and TDM, these models enable individualized dose adjustment [5]. Recent clinical evidence highlights the growing role of MIPD in optimizing vancomycin therapy across special populations. For instance, Hall et al. demonstrated that AUC-guided dosing using Bayesian MIPD significantly improved safety outcomes, with reduced incidence of vancomycin-associated acute kidney injury and shorter hospital stay compared to traditional trough-guided dosing [9]. These studies collectively support the integration of validated PopPK models and MIPD platforms to enhance dosing precision and clinical outcomes in critically ill or renally impaired patients [10]. PopPK modeling is a powerful tool for dose individualization and regimen selection within specific patient groups. Such models allow estimation of typical PK parameters and identification of covariates (e.g., age, body weight, CRRT intensity, albumin concentration) that influence drug disposition [8,11]. Despite its clinical importance, only a limited number of PopPK studies have investigated vancomycin PK and dosing during CRRT, and their recommendations vary considerably.

To our knowledge, no systematic review to date has comprehensively synthesized these PopPK studies in critically ill adult patients undergoing CRRT. Existing reports are restricted to single-center PopPK analyses or dosing simulations, without an integrated appraisal of PK parameters, CRRT covariates, and dose recommendations. This study aims to systematically review PopPK studies of vancomycin in critically ill patients undergoing CRRT, summarizing PK parameters, covariates, and proposed dosing strategies. By doing so, we seek to provide clinicians with an evidence-based overview to support individualized vancomycin dosing during CRRT.

## 2. Materials and Methods

This systematic review was conducted in accordance with the PRISMA (Preferred Reporting Items for Systematic Reviews and Meta-Analysis) 2020 guidelines. The protocol was registered in the International Prospective Register of Systematic Reviews (PROSPERO, registration number CRD420250655157, registration date 24 February 2025).

### 2.1. Inclusion Criteria

Studies were included if they reported PopPK analyses of vancomycin in adult critically ill patients receiving CRRT. Eligible studies had to provide PK parameters (e.g., clearance, volume of distribution, half-life, AUC) and/or dosing recommendations. Both parametric and non-parametric PopPK modeling approaches were eligible for inclusion. Only full-text articles published in English were considered. Exclusion criteria were case reports or case series without PopPK modeling, pediatric-only studies, in vitro or animal experiments, reviews, editorials, and conference abstracts.

### 2.2. Search Strategy

A comprehensive literature search was conducted in PubMed/MEDLINE, Embase, Web of Science, and the Cochrane Library, covering all records from inception to 28 February 2025. The search strategy combined MeSH/Emtree terms and free-text keywords related to “vancomycin,” “population pharmacokinetics,” and “continuous renal replacement therapy” (CRRT, CVVH, CVVHD, CVVHDF). An example PubMed search string is provided in the Supplementary Materials. Reference lists of eligible studies and relevant reviews were also screened. Only peer-reviewed full-text research articles in English were considered.

### 2.3. Selection of Studies

**Figure 1:**
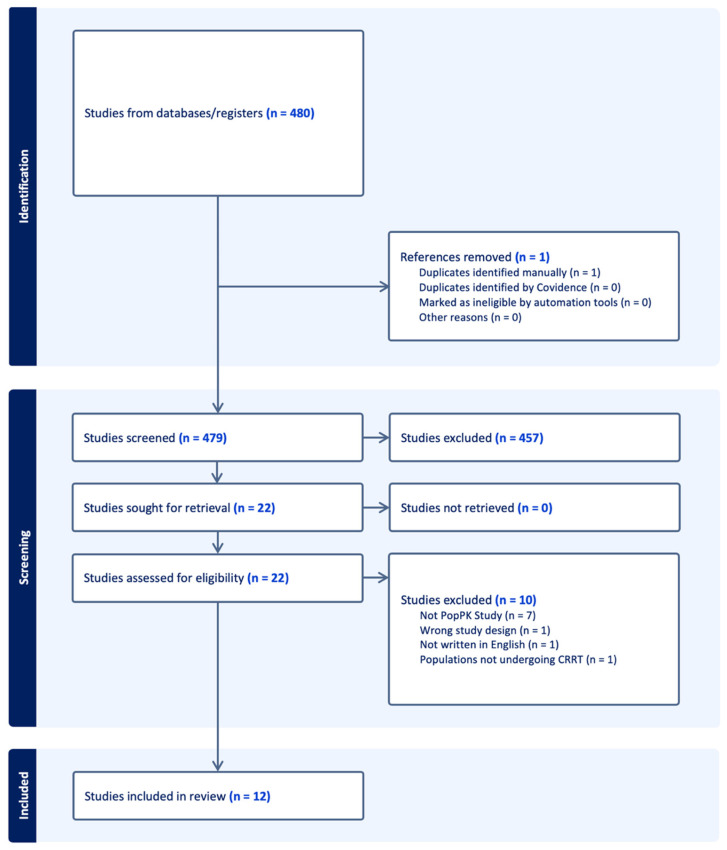
PRISMA 2020 flow diagram summarizing the selection process of studies included in the systematic review. Note: The flow diagram was created automatically in Covidence (Veritas Health Innovation, Melbourne, Australia; available at www.covidence.org (accessed on 16 October 2025) based on study selection and exclusion data entered by reviewers. Adapted in accordance with the PRISMA 2020 statement [12].

All retrieved records were imported into Covidence systematic review software (Veritas Health Innovation, Melbourne, Australia; updated 4 July 2023). Two reviewers (NS and SM) independently screened titles and abstracts according to the eligibility criteria. Full texts of potentially relevant studies were then assessed. Disagreements were resolved through discussion or consultation with a third reviewer (NY). The PRISMA flow diagram of the study selection process is presented in Figure 1.

### 2.4. Data Extraction

Data extraction was performed independently by two reviewers using a standardized form within Covidence. Discrepancies were resolved through discussion or by consultation with a third reviewer. Extracted information was organized into three main domains to reflect the specific focus of this review:

- Study characteristics
  o Author, year of publication;
  o Study design;
  o Number of patients included and demographic information;
  o PK analysis method;
  o Analysis software;
  o Clinical setting.
- Dose and CRRT modality data
  o Loading and maintenance dosing regimens, including infusion type;
  o Dose adjustment methods (e.g., based on effluent rate, body weight, TDM);
  o CRRT modality and parameters.
- PK findings and dosing recommendations
  o Reported PK parameters;
  o Identified covariates influencing PK parameters;
  o Clinical outcomes when reported;
  o Final dose recommendations proposed by the study authors.

As no universally accepted standardized appraisal framework exists for PopPK model development studies, a structured descriptive methodological review was performed, informed by the ClinPK (Reporting Guidelines for Clinical Pharmacokinetic Studies) reporting statement [13]. Extracted domains included study design, sample size, PK analysis method, covariates evaluated, structural model characteristics, and model validation approaches.

### 2.5. Methodological Appraisal of Included PopPK Studies

Given the absence of a validated risk-of-bias instrument specifically designed for PopPK model development studies, no standardized risk-of-bias tool was applied. Instead, a structured descriptive methodological appraisal was conducted, focusing on study design, structural model specification, covariate modeling strategy, validation procedures, and reporting transparency, informed by established pharmacometric reporting recommendations. As PopPK studies aim to estimate pharmacokinetic parameters rather than comparative treatment effects, classification into hierarchical risk categories was not methodologically appropriate. Due to heterogeneity in structural models, CRRT modalities, and covariate parameterization, results were synthesized narratively rather than quantitatively pooled.

To minimize the influence of structural model misspecification or covariate overfitting on the narrative synthesis, predefined methodological domains were systematically extracted, including structural model type, covariate selection strategy, internal and external validation procedures, and reporting transparency. No quantitative weighting or scoring system was applied; instead, methodological characteristics were presented descriptively to allow readers to independently appraise model credibility.

### 2.6. Reporting Bias Assessment

Formal assessment of reporting bias (e.g., publication bias or selective outcome reporting) was not performed. PopPK model development studies do not report comparative effect estimates suitable for funnel plot-based or statistical asymmetry analyses. Moreover, the primary outcomes of interest (model structure, clearance estimates, covariate inclusion) are methodological parameters rather than effect sizes. Therefore, conventional approaches to assess reporting bias were not applicable to this review.

### 2.7. Certainty of Evidence Assessment

A formal certainty-of-evidence assessment (e.g., GRADE) was not conducted. GRADE methodology is primarily designed for intervention studies evaluating comparative effectiveness or clinical outcomes. The included studies in this review were PopPK model development studies aimed at parameter estimation and covariate identification, without pooled effect estimates or comparative clinical endpoints. Therefore, application of GRADE was not methodologically appropriate.

## 3. Results

The initial search identified 480 records. After removal of one duplicate, 479 studies remained for title and abstract screening. Of these, 457 were excluded at the screening stage. A total of 22 full-text articles were assessed for eligibility, and 10 were excluded (7 not PopPK studies, 1 with wrong study design, 1 not written in English, and 1 including populations not undergoing CRRT). Finally, 12 studies met the inclusion criteria and were included in the systematic review (Figure 1).

### 3.1. Study Characteristics and Methodological Features

A total of 12 PopPK studies evaluating vancomycin in critically ill adult patients undergoing CRRT were included. These studies were published between 2013 and 2023. Study design varied: four studies were prospective, while eight were retrospective or retrospective with mixed components (e.g., external validation, Bayesian evaluation, database analysis). Sample sizes ranged widely, from 9 to 159 patients, with several studies also reporting the number of PK samples collected. Patient age was generally in the middle-aged to elderly range (mean or median values between 55 and 69 years). Residual diuresis was usually absent or markedly reduced; in some studies, anuric or oliguric patients predominated, while others explicitly excluded patients with preserved urine output. Overall, the included studies demonstrated methodological and clinical heterogeneity in terms of design, patient population, and CRRT settings (Table 1).

### 3.2. Vancomycin Dosing Practices and CRRT Modalities

Loading and maintenance dosing strategies for vancomycin during CRRT were highly heterogeneous across the included studies. Reported loading doses ranged from 16 to 35 mg/kg, with some studies recommending fixed doses (e.g., 2 g) and others reporting weight-based regimens. Several investigations did not standardize loading dose administration, leaving the decision to clinicians. A few studies performed simulated dose optimization, suggesting higher loading doses (≥25–30 mg/kg) to achieve early target attainment.

Maintenance dosing also varied markedly. Regimens included intermittent administration of 750–1500 mg every 12 h, fixed doses of 1000 mg/day, weight-based approaches (8–20 mg/kg/day), and continuous infusions (typically 14–20 mg/kg/day). In continuous-infusion studies, serum concentrations were maintained within the target range of 20–30 mg/L. Notably, some studies proposed individualized adjustments using Bayesian modeling or TDM-guided dose modifications. Infusion practices differed, with the majority employing intermittent infusion (typically 1–2 h), while others used continuous infusion or reported both strategies in simulation analyses.

Among the CRRT modalities evaluated across the included studies, CVVH and CVVHDF were the most frequently represented approaches, whereas CVVHD and CHDF were less commonly investigated. One study specifically examined high-volume hemofiltration (HVHF). At the study level, two investigations were restricted to CVVH (including HVHF), and two were conducted exclusively in CVVHDF/CHDF cohorts. Three additional studies predominantly included CVVHDF populations (>90% of patients), while four incorporated mixed CRRT modalities within the same model. In three studies, CRRT modality was not clearly specified.

Across modalities, effluent rates ranged from approximately 26 to 123 mL/kg/h, with some studies explicitly modeling effluent rate as a covariate for vancomycin clearance. Dialysate and replacement flow rates were less consistently reported and, when available, showed broad variation (e.g., dialysate 500–1400 mL/h; replacement 200–1500 mL/h). Other CRRT-related parameters, including filter type and anticoagulation strategy, were inconsistently described.

Filter types (e.g., polyacrylonitrile, polysulfone, or polymethyl methacrylate membranes) were heterogeneous, and no clear association with PK outcomes was observed. Anticoagulation strategies were variably applied, most commonly unfractionated heparin or regional citrate, but rarely standardized. Dose adjustment methods differed widely, including TDM-based approaches, model-informed simulations, Bayesian feedback, and Monte Carlo simulations.

Overall, the findings highlight the absence of standardized dosing strategies for vancomycin during CRRT, with substantial heterogeneity in loading and maintenance regimens, infusion practices, and CRRT intensity, alongside inconsistent reporting of filter type, anticoagulation, and dose adjustment methodology. This heterogeneity underscores the challenge of translating study findings into uniform clinical dosing recommendations (Table 2).

### 3.3. Pharmacokinetic Findings and Dose Recommendations

Across the included studies, vancomycin PK during CRRT demonstrated pronounced heterogeneity. Reported clearance values ranged widely, from as low as 0.7 L/h to over 3.0 L/h, reflecting the substantial influence of extracorporeal and patient-specific factors. Effluent rate consistently emerged as a major determinant of drug clearance, but residual urine output, vasopressor use, and CRRT modality or intensity also contributed significantly. Across the included studies, residual diuresis was generally operationalized using 24-h urine output, either as a continuous covariate or categorized (e.g., anuric vs. non-anuric). Formal measurement of native creatinine clearance was rarely reported in patients undergoing CRRT. Similarly, estimates of the volume of distribution spanned from 0.8 L/kg to more than 100 L, underscoring the profound variability in fluid status, capillary leak, and disease severity across critically ill cohorts. While half-life was inconsistently reported, available estimates ranged between 6 and 20 h, with outliers extending up to 25–60 h in patients with markedly reduced clearance, expanded volume of distribution, or both. Drug exposure, expressed as AUC24, showed comparable variability, ranging between 319 and 652 mg·h/L, with pharmacodynamic targets (AUC24/MIC ≥ 400) consistently achieved only when regimens were individualized and supported by TDM (Table 3). With respect to dosing implications, the available evidence consistently supports the use of an initial loading dose in the range of 20–30 mg/kg to achieve therapeutic exposure more rapidly. Maintenance dosing, however, varied substantially and was most strongly influenced by effluent rate and CRRT intensity. Commonly suggested strategies included weight-based regimens of 15–20 mg/kg every 24–48 h or fixed doses of 750–1000 mg every 12 h, though these regimens were generally effective only when adjusted to effluent rates exceeding 25–35 mL/kg/h. Several authors proposed continuous infusion or Bayesian-guided approaches as alternatives to improve target attainment and minimize variability. Overall, the evidence indicates that standardized regimens are insufficient in the CRRT setting, and that individualized dosing guided by effluent flow, patient-specific covariates, and TDM is required to ensure efficacy and safety (Table 3). To enhance transparency of model structure, all covariates explored and statistically confirmed across the included PopPK studies were systematically summarized in Table 4. Clinical outcomes were inconsistently reported across the included studies. None of the studies provided data on nephrotoxicity rates. Mortality was reported with substantial variation, ranging from 23% in Bayesian TDM cohorts to 60% in control groups, although differences in outcome definitions limited comparability.

### 3.4. Methodological Characteristics of Included PopPK Models

The methodological characteristics of the included PopPK studies are summarized in Table 5. Model structures varied across studies, including both one- and two-compartment approaches, implemented using different estimation algorithms (e.g., FOCE-I, SAEM, nonparametric methods). Covariate selection strategies were predominantly based on stepwise likelihood ratio testing, although exploratory or biologically driven approaches were also reported. Internal validation procedures were variably applied. Most studies presented goodness-of-fit plots. Predictive checks (VPC/pcVPC/NPC) were performed in 8 of 12 studies, and bootstrap resampling was reported in 8 of 12 studies. External validation using an independent dataset was reported in 3 of 12 studies. Reporting of CRRT operational parameters, sampling schedules, and dosing documentation differed across studies.

## 4. Discussions

To our knowledge, this is the first systematic review to synthesize PopPK studies of vancomycin in critically ill patients undergoing CRRT. A total of 12 studies were included, covering a wide range of study designs, sample sizes, and CRRT modalities. Despite this heterogeneity, several descriptive patterns were observed across independently developed models. Across studies, patient age was generally clustered around 60 years, suggesting that age itself is unlikely to act as a major source of PK variability in this setting.

Effluent rate was the most consistently retained covariate influencing clearance across independently developed PopPK models. At conventional intensities (20–25 mL/kg/h), maintenance regimens of approximately 15–20 mg/kg/day or 5 mg/kg every 12 h were sufficient to achieve therapeutic exposure. In contrast, higher effluent rates (25–45 mL/kg/h) were associated with increased clearance and prompted evaluation of more aggressive dosing strategies, such as 7.5 mg/kg every 12 h or 20–25 mg/kg/day administered as continuous infusion. High-volume hemofiltration (≥70 mL/kg/h) necessitated markedly higher doses, with some models exploring doses up to 1–1.5 g every 12 h. Residual urine output was also consistently associated with reduced dose requirements, with patients maintaining diuresis often requiring approximately 30–50% lower doses compared with anuric patients [1,2,3,5,8,14,15,16,17,18,19,20]. As summarized across studies (Table 2, Table 3 and Table 4), effluent rate and residual urine output were the most consistently retained covariates across the included PopPK models, confirming their predominant influence on vancomycin clearance. In contrast, other variables such as serum albumin or hemodynamic status showed inconsistent associations and were identified as significant only in isolated studies. Although hypoalbuminemia has been associated with increased free drug fraction and higher extracorporeal clearance in some analyses [19], this finding was not consistently reproduced across PopPK studies and should therefore be interpreted with caution.

The recurrent retention of effluent rate should also be interpreted in light of potential collinearity and covariate competition. In CRRT populations, effluent intensity, ultrafiltration rate, albumin concentration, vasopressor use, and hemodynamic instability are often physiologically interrelated. When stepwise covariate selection strategies are applied in relatively small datasets, correlated variables may compete for inclusion, resulting in retention of the statistically dominant predictor while other clinically relevant factors are excluded. Therefore, the apparent dominance of effluent rate likely reflects both its true contribution to extracorporeal clearance and the dynamics of model-building strategies rather than exclusive mechanistic causality.

The interpretation of effluent rate as the dominant clearance predictor should be considered in the context of CRRT modality distribution. Only a minority of studies were restricted to a single modality, whereas several incorporated mixed CRRT modalities or predominantly CVVHDF cohorts. In convective modalities such as CVVH/HVHF, ultrafiltration rate directly determines solute removal, while in combined modalities (CVVHDF/CHDF), total effluent flow reflects both convective and diffusive clearance components. Because most included models parameterized extracorporeal intensity using total effluent flow rather than modality-specific clearance terms, effluent rate may function as a composite indicator of CRRT intensity across heterogeneous settings. Consequently, modality-specific contributions to vancomycin clearance cannot be clearly delineated based on the currently available PopPK evidence.

Heterogeneity in the definition and inclusion criteria of residual diuresis across studies may have introduced spectrum restriction. Several models were developed in predominantly anuric or severely oliguric populations, while others excluded patients with preserved renal function. In such settings, the limited variability in native renal clearance may reduce the detectable contribution of residual kidney function to total clearance, potentially leading to greater attribution of variability to CRRT-related parameters such as effluent rate. Consequently, PopPK models derived from highly selected anuric cohorts may demonstrate reduced transportability to real-world CRRT populations characterized by heterogeneous and dynamically evolving renal recovery trajectories. These considerations further support cautious model application and the continued reliance on TDM in patients with changing renal function.

Importantly, the variability observed across studies reflects not only patient-level heterogeneity but also differences in structural model assumptions, covariate selection strategies, and CRRT parameterization. By systematically comparing these modeling approaches, this review identifies how variability has been operationalized within PopPK frameworks rather than merely documenting clinical diversity among ICU populations.

The wide range of reported vancomycin clearance values (0.7–3.0 L/h) likely reflects differences in CRRT intensity, patient characteristics, and modeling strategies. Given differences in study design, CRRT parameter reporting, sampling density, and covariate modeling approaches, direct comparison of clearance magnitudes across studies should be performed cautiously. Variability in reported estimates may reflect both biological heterogeneity and differences in methodological implementation.

In addition to clearance variability, dispersion in reported Vd (including values > 100 L) and occasional prolonged half-life estimates warrants cautious interpretation. Severe capillary leak, fluid resuscitation, hypoalbuminemia, and positive fluid balance in CRRT patients may physiologically expand distribution of hydrophilic drugs such as vancomycin. However, small cohorts, sparse sampling in two-compartment models, and limited covariate characterization may also contribute to parameter inflation. Therefore, extreme values should be interpreted within both physiological and methodological contexts.

From a pharmacometric perspective, variability was typically partitioned into interindividual variability (IIV) in clearance and volume parameters and residual unexplained variability reflecting intra-individual and measurement-related factors. Across studies, IIV in vancomycin clearance was frequently substantial, indicating that CRRT intensity alone does not fully account for between-patient differences. In contrast, intra-individual variability was captured through residual error models and differed according to sampling density and assay characteristics. This review did not attempt to quantitatively compare variance components (omega or sigma estimates) across models, as structural differences precluded formal synthesis; however, the consistent reporting of considerable IIV underscores the need for individualized dosing supported by TDM.

The importance of an adequate loading dose was also highlighted across the included studies. Loading doses in the range of 15–30 mg/kg, and up to 35 mg/kg at higher effluent rates, were frequently evaluated or proposed within individual PopPK models to facilitate early attainment of pharmacodynamic targets [1,2,5,8,15,19]. Model-based simulations suggested that lower loading doses may delay attainment of target AUC/MIC values. These observations align with broader evidence demonstrating that extracorporeal therapies substantially alter the pharmacokinetics of hydrophilic agents such as vancomycin through changes in volume of distribution and clearance [21], underscoring the importance of early TDM.

The mode of infusion also deserves consideration. While continuous infusion may theoretically provide more stable exposures and lower daily dose requirements, practical barriers such as limited vascular access and potential drug incompatibilities restrict its use in the ICU. As a result, most included studies focused on intermittent infusion, and dosing recommendations largely reflect this practice.

When compared with international guidelines, such as the American Society of Health-System Pharmacists (ASHP) recommendations (20–25 mg/kg loading and 7.5–10 mg/kg q12 h at effluent rates of 20–25 mL/kg/h), our findings both support and extend current guidance. Specifically, effluent rate consistently proved to be the most reliable predictor of clearance, and higher-than-guideline dosing strategies were often explored at effluent rates ≥ 25–45 mL/kg/h [22]. In addition, patient-specific factors such as residual diuresis and albumin—although not explicitly incorporated into existing guideline algorithms—were examined as potential modifiers of dosing requirements in several models.

Taken together, the recurring evidence patterns observed across the included PopPK studies—including the dominant role of effluent rate, the modifying effect of residual diuresis, and the variability in loading and maintenance dose requirements—are synthesized in Box 1. This synthesis represents a structured summary of model-derived findings and does not constitute pooled quantitative analysis or prescriptive dosing guidance.

In this context, the model-based meta-analytic work by Claisse et al. [23] provides an important complementary perspective. By integrating pharmacokinetic data across multiple renal replacement therapy modalities, their analysis quantitatively demonstrated that vancomycin clearance increases with extracorporeal clearance intensity and that dosing requirements cannot be reliably extrapolated across heterogeneous RRT settings. These findings are concordant with the evidence patterns summarized in Box 1 and reinforce the conclusion that simplified or uniform dosing schemes are unlikely to perform adequately during CRRT, highlighting the need for individualized dosing strategies informed by pharmacokinetic modeling and TDM.

Box 1 Key clinical signals * emerging from recurrent PopPK modeling patterns.

- Effluent intensity as a primary determinant: Effluent rate was the most consistently retained predictor of vancomycin clearance across models. Higher effluent intensities were reproducibly associated with increased clearance.
- Loading dose: Loading doses of 25–30 mg/kg were most commonly evaluated to achieve early target attainment, with higher doses explored at increased effluent rates.
- Maintenance dose variability: Maintenance requirements varied substantially and were closely linked to CRRT intensity. Fixed regimens performed inconsistently across heterogeneous settings.
- Residual diuresis modifies clearance: When retained, residual urine output was associated with reduced clearance. Definitions were heterogeneous and primarily based on 24-h urine volume.
- TDM remains essential: Substantial interindividual variability persisted across models, supporting early and repeated TDM.

* These statements summarize recurrent modeling patterns across heterogeneous PopPK studies and are intended to enhance clinical interpretability. They do not constitute prescriptive dosing algorithms, and individualized dosing supported by TDM remains essential.

Recent clinical evidence further underscores the potential of integrating MIPD into CRRT settings to refine vancomycin therapy. Hall et al. demonstrated that Bayesian AUC-guided dosing, even when based on single-sample monitoring, significantly reduced vancomycin-associated acute kidney injury and shortened both hospital and ICU length of stay compared with trough-guided regimens [9]. These findings support the transition from empiric or nomogram-based CRRT dosing toward adaptive Bayesian-assisted approaches that dynamically incorporate patient-specific covariates and real-time TDM data. Future multicenter investigations integrating PopPK, pharmacodynamic targets, and clinical outcomes are warranted to confirm whether MIPD-based strategies can consistently improve efficacy and safety in critically ill patients undergoing CRRT.

This review does not attempt to statistically resolve inter-study heterogeneity or quantify effect sizes across models. Given differences in structural models, sampling design, covariate parameterization, and reporting practices, direct pooling of clearance or volume estimates was not methodologically appropriate.

Structural identifiability and model complexity warrant careful consideration in this context. Several included studies implemented two-compartment models despite relatively small sample sizes and limited sampling density. In such settings, reliable estimation of peripheral volume and intercompartmental clearance parameters may be challenged by practical non-identifiability, particularly when early distribution-phase concentrations are sparse. Although most studies reported acceptable goodness-of-fit diagnostics and, in many cases, bootstrap or predictive checks, formal assessment of structural identifiability was not described.

Risk of overparameterization should also be acknowledged. Stepwise covariate modeling strategies were widely applied, often exploring multiple CRRT-related variables (e.g., effluent rate, ultrafiltration rate, residual diuresis, albumin). In smaller cohorts, the ratio between the number of estimated parameters and the number of subjects may increase the risk of overfitting, potentially limiting external generalizability. Only a minority of studies performed external validation, which restricts assessment of model transportability across centers.

Shrinkage and sampling design considerations further influence interpretability. Reporting of eta- and epsilon-shrinkage was inconsistent, precluding systematic evaluation of individual parameter reliability. High shrinkage, particularly in sparsely sampled datasets, may attenuate the apparent strength of covariate–parameter relationships and limit the robustness of Bayesian post hoc estimates used for MIPD. Moreover, sampling schedules varied substantially across studies, and limited early post-dose sampling in some cohorts may have constrained accurate characterization of distribution kinetics.

These methodological factors do not invalidate the included models but should be considered when interpreting between-study variability and when translating model-derived dosing recommendations into clinical practice.

Although no formal model credibility scoring framework was applied, the structured extraction of model development and validation characteristics was intended to enhance transparency and reduce the risk that inadequately validated models disproportionately influenced the synthesis. Nevertheless, heterogeneity in reporting practices across studies limits the ability to formally compare model robustness.

This study has several important limitations. First, the number of available PopPK studies remains limited, and most included relatively small patient cohorts, restricting the robustness of model-based inferences. Second, there was substantial heterogeneity across studies with respect to CRRT modalities, effluent rates, filter membranes, anticoagulation strategies, and modeling approaches, which markedly limits the ability to derive a unified dosing algorithm applicable to all clinical settings. In the absence of formal effect size quantification or statistical heterogeneity analysis, the dosing recommendations synthesized in this review should be interpreted as descriptive model-derived patterns rather than transportable algorithms across all CRRT platforms. Differences in diffusive–convective balance, membrane adsorption characteristics, circuit downtime, and anticoagulation protocols may alter effective drug clearance in ways not uniformly captured across individual PopPK studies. Moreover, no formal model credibility grading framework was applied; instead, validation characteristics were descriptively extracted to enhance transparency. Therefore, extrapolation of dosing strategies across heterogeneous CRRT settings should be performed cautiously and supported by local TDM. As highlighted by Kanji et al. [24], drug disposition during CRRT is influenced by multiple interacting factors, including the balance between diffusive and convective clearance, effluent flow rate, membrane adsorption properties, and the use of citrate versus heparin anticoagulation, all of which may variably alter antimicrobial clearance over time. Variability in filter type and surface area further complicates interpretation, as adsorption-related drug loss may be clinically relevant for some antimicrobials, including vancomycin, particularly during the early phase of CRRT. In addition, differences in anticoagulation strategies may indirectly affect filter lifespan, circuit downtime, and effective drug clearance, introducing additional pharmacokinetic variability. Third, most included studies primarily focused on pharmacokinetic endpoints and did not report clinically meaningful outcomes such as mortality, infection resolution, or nephrotoxicity, precluding assessment of how proposed dosing strategies translate into clinical efficacy or safety. Together, these limitations underscore the challenges of generalizing any single dosing recommendation and emphasize the need for individualized dosing approaches that integrate patient-specific factors, CRRT characteristics, and TDM [24].

Despite these limitations, this systematic review provides a structured descriptive synthesis for interpreting vancomycin dosing patterns during CRRT. Across included PopPK studies, loading doses of approximately 25–30 mg/kg (up to 35 mg/kg at higher effluent rates) were commonly evaluated, maintenance dosing varied according to CRRT intensity and residual diuresis, and early and repeated TDM was consistently emphasized to support target attainment. Future research should prioritize prospective, multicenter PopPK studies with standardized outcome reporting to bridge the gap between PK findings and clinical efficacy.

## 5. Conclusions

Across included PopPK studies of vancomycin during CRRT, effluent rate was the most frequently retained covariate and was consistently incorporated as a predictor of clearance. Residual urine output and serum albumin were retained in a smaller number of models, reflecting variability in covariate selection and model structure across studies.

Given the heterogeneity in study design, CRRT modalities, and modeling approaches, these findings represent patterns of covariate inclusion rather than pooled quantitative effect estimates. No formal assessment of inter-study reproducibility or effect magnitude was performed.

Overall, current evidence supports the integration of CRRT intensity and patient-specific factors into model-informed dosing strategies, with TDM remaining essential to individualize therapy in this highly variable population.

## Figures and Tables

**Table 1:** Characteristics of included population pharmacokinetic studies.

Author, Year	Study Design	PK Analysis Method	Analysis Software	Patients/Samples	Age (Year)	Residual Diuresis
Beumier, 2013 [1]	Prosp	2-comp; NLME	NONMEM v6.1 (FOCEI, ADVAN3, modeling); PsN, R, Xpose (support); bootstrap, VPC (validation)	32/NR	55 (47–64)	NR
Garreau, 2021 [2]	Retro; external validation	2-comp; NLME (SAEM alg.)	Monolix 2020R1 (modeling); Simulx (simulation)	162/NR; Cohorts: L78/V84; CRRT: 26	L: 68.9 ± 12.3; V: 58.9 ± 15.3	Anuria assumed (CRCL = 0)
Oda, 2020 [3]	Retro modelling; Prosp Bayesian evaluation	2-comp	NONMEM v7.3 (modeling; FOCEI); PsN/R (support)	17/80; Prosp eval: 23	64 (19–92)	RUO < 0.5 mL/kg/h in 82%
Udy, 2013 [5]	Retro	1-comp; zero-order input	NONMEM v6.1 (modelling; FOCEI)	81/199	61.0 ± 15.6	Excluded if >500 mL/day
Kirwan, 2021 [8]	Retro; single centre	1-comp; NPAG	PMetrics (R package)	24/155	65.5 ± 12.3	2.5 mL/24 h (IQR 0–92.5; 0–1836)
Bae, 2019 [14]	Retro	2-comp; FO elimination	NONMEM v7.4 (FOCEI, modeling); RStudio v0.99, R v3.2.2, Xpose4 v4.5.3 (support)	9/NR	NR	NR
Escobar, 2014 [15]	Prosp	2-comp; NLME	NONMEM v7.2 (modeling); bootstrap; Prism/Excel (support)	9/NR	57 ± 14	Anuric (n = 6); Oliguric (n = 3)
Lin, 2021 [16]	Prosp; external validation	1-comp; NLME (EM alg.)	Kinetica 4.4.1.	466 total; 374 PopPK; CRRT 87 (23.3%)	NR (overall cohort 62 (18–93)	NR (SCr included as covariate)
Wang, 2023 [17]	Retro modeling; MIMIC-IV	1-comp	NONMEM v7.2	159/1186	64 (52–72)	NR
Wang, 2021 [18]	Prosp	2-comp; FO elimination	NONMEM v7.3 (modeling); PsN/R (support)	11/131	63 (20–83)	Mostly anuric/oliguric; median 300 mL/day
Yamazaki, 2020 [19]	Retro	2-comp; NLME	Phoenix NLME v1.4 (WinNonlin v6.4)	25/130	65 (21–83)	UO: 116 mL/day (0–2913)
Yu, 2023 [20]	Retro, multicenter	1-comp; FO elimination	NONMEM v7.5 (modeling); PDxPop v5.3.1, R (support)	71/113	61.6 ± 14.6	UO: 160 mL/day (IQR 7–780; max 6220)

Retro: Retrospective; Prosp: Prospective; CRCL: Creatinine Clearance; 1-comp: one-compartment model; 2-comp: two-compartment model; NLME: nonlinear mixed-effects modeling; NPAG: nonparametric adaptive grid; FO: first-order; EM alg.: expectation–maximization algorithm; L: learning cohort; V: validation cohort; CRRT: continuous renal replacement therapy; EV: external validation; Prosp eval: Prospective evaluation; IQR: Interquartile Range; NONMEM: Nonlinear Mixed-Effects Model; NR: Not Reported; PopPK: Population Pharmacokinetic; RUO: Reduced Urine Output; SCr: Serum creatinine; UO: Urinary Output.

**Table 2:** Vancomycin dosing strategies and CRRT settings.

Author, Year	Loading Dose	Maintenance Dose	Infusion Type	CRRT Modality	Effluent Rate	Replacement/Dialysate Flow
Beumier, 2013 [1]	35 mg/kg (median 2750 mg; 2250–3150), 4 h	14 mg/kg/day (median 1100 mg), CI	Continuous	CVVHDF/CVVH	34 mL/kg/h (IQR 25–43)	Dialysate 1400 (0–1650); UF 1500 (1500–2000) mL
Garreau, 2021 [2]	22.7 ± 7.5 mg/kg (IBW)	28.6 ± 9.4 mg/kg	Continuous	NR	35.6 ± 18.7 mL/min (L); 28.7 ± 6.5 mL/min (V) (~30–37 mL/kg/h)	NR
Oda, 2020 [3]	20 mg/kg in 2 pts (22–23)	8.7 mg/kg/day (3.4–21.0)	Intermittent	CVVHDF 15 (88%); CVVHD 2 (12%)	11.7 mL/kg/h (10.1–50.3)	NR
Udy, 2013 [5]	16.4 ± 5.5 mg/kg (ABW)	23.7 ± 8.1 mg/kg/day, CI	Continuous	CVVH 41 (50.6%); CVVHDF 40 (49.4%)	30.8 ± 13.1 mL/kg/h	CVVH: UF only; CVVHDF: UF + Dial (Dial: 19.4 ± 5.8 mL/kg/h) (UF: 21.2 ± 7.2 mL/kg/h)
Kirwan, 2021 [8]	25 mg/kg	15–20 mg/kg q24 h	Intermittent (10 mg/min)	CVVHDF	Variable; NR	Q_eff_ = Q_dial_ + Q_rep_ + UF + PBP
Bae, 2019 [14]	NR	1 g q24 h	Intermittent (1 h)	NR	NR	NR
Escobar, 2014 [15]	1 g IV over 1 h	1 g q24 h (1-h infusion)	Intermittent (2 h); Continuous simulated	HVHF (pre-dilution)	100 ± 18 mL/kg/h (range 69–123)	Pre-dilution, substitution fluid ≈100 mL/kg/h
Lin, 2021 [16]	Variable (0.5–1 g, q6–24 h; clinician decision)	Same as LD; clinician-adjusted	Intermittent	NR	NR (categorical only)	NR
Wang, 2023 [17]	NSR (median initial dose 1 g/day)	1000 mg/day (q24 h common; some q12 h)	Intermittent	CVVHDF 96.1%; few CVVH/CVVHD/SCUF	29.4 mL/kg/h (IQR 26.0–32.5)	Dialysate ~900; Pre ~1500; Post ~200 mL/h
Wang, 2021 [18]	Empirical (0.5 g q8–24 h)	Empirical (0.5 g q8–24 h) Clinician-adjusted; simulated regimens	Intermittent	CVVH	UFR: 33.3 mL/kg/h (range 18–39)	NR
Yamazaki, 2020 [19]	27.1 mg/kg (optimal, simulated); tested 50 mg/kg	9.7 mg/kg q24 h (optimal, sim.); tested 14 mg/kg	Intermittent	CHDF (PMMA filter)	26.3 ± 6.3 mL/kg/h	Dialysate 500–1000 mL/h; supplement 300–500 mL/h
Yu, 2023 [20]	NR	500–3000 mg/day (median 1000; 15.4 mg/kg)	Intermittent	CVVH 40 (56.3%); CVVHDF 31 (43.7%)	NR	NR

ABW: Actual Body Weight; CHDF: Continuous Hemodiafiltration; CRRT: Continuous Renal Replacement Therapy; CVVH: Continuous Veno-Venous Hemofiltration; CVVHDF: Continuous Veno-Venous Hemodiafiltration; HVHF: High-Volume Hemofiltration; IBW: Ideal Body Weight; IQR: Interquartile Range; LD: Loading Dose; NR: Not Reported; PBP: pre-blood pump; PMMA: polymethyl methacrylate; Qdial: Dialysate flow rate; Qeff: Effluent flow rate; Qrep: Replacement rate; UF: Ultrafiltration.

**Table 3:** Pharmacokinetic parameters and clinical outcomes.

Author, Year	CL	V_D_ (V_C_, V_P_)	AUC_24_	C_MIN_	C_MAX_	Dose Recommendations & Covariates
Beumier, 2013 [1]	1.99 L/h (95% CI 1.80–2.02)	V_c_ = 35.8 L; V_p_ = 63.2 L	652 (596–789) mg·h/L	NR	NR	Dose: LD 35 mg/kg; MD 14 mg/kg/day (CI); TDM required; not sufficient if MIC ≥ 2 Covariates: TBW on V1; CRRT intensity on CL
Garreau, 2021 [2]	0.79 L/h (modeled on effluent)	V_c_ = 27.3 L; V_p_ = 61.3 L;	Day 2 AUC24–48: 530 ± 160 (L); 515 ± 341 (V) mg·h/L	NR	NR	Dose: LD 27.5 mg/kg (IBW, 2 h); MD 17.5–20 mg/kg/day (effluent 20–30 mL/min); alternative 25–27.5 mg/kg/day; AUC-guided TDM mandatory Covariates: IBW on V1; CRCL on CL (no CRRT); CRRTEFR on CL (CRRT)
Oda, 2020 [3]	2.12 → 0.34 L/h (RUO+); linear with effluent (0.672 × rate)	91.3 L/70 kg	NR (Bayesian posterior possible)	10–20 mg/L target; attainment 87% (Bayes-TDM) vs. 53.8%	NR	Dose: RUO−: 12–23 mg/kg q12 h; RUO+: 3–11 mg/kg q12 h (≤50% of RUO−) Covariates: RUO & effluent rate; Bayes-TDM improved target attainment
Udy, 2013 [5]	2.9 L/h (IQR 2.4–3.4); BSV 34.7%	0.8 L/kg (IQR 0.6–1.1); BSV 49.8%	NR	24.6 ± 9.2 mg/L (day 1 mean)	NR	Dose: LD 15 mg/kg; CI targeting 20–30 mg/L (~70% reached at 24 h); no standardized MD, TDM required Covariates: None
Kirwan, 2021 [8]	2.59 ± 0.49 L/h (base);	80.98 ± 16.9 L	PTA ~100% (AUC/MIC ≥ 400, MIC 1; 2 g LD + 750 mg q12 h)	15.7 ± 5.1 mg/L	29.1 ± 7.5 mg/L	Dose: LD 2 g; MD 750 mg q12 h; AUC-guided TDM recommended Covariates: Effluent rate & vasopressor use
Bae, 2019 [14]	0.716 L/h	NR	Higher vs. non-CRRT	NR	NR	Dose: NSR Covariates: CLcr, CRRT on CL; WT on V2
Escobar, 2014 [15]	2.7–2.9 L/h	V_c_ = 11.8–11.9 L; V_p_ = 17.3–18.0 L	319 ± 251 mg·h/L (0–12 h)	12.2 ± 10.6 mg/L (12 h; mostly <11)	72.7 ± 53.9 mg/L	Dose: LD ≥ 20–30 mg/kg (2 h); MD 750–1500 mg q12 h (HVHF-dependent); CI 1000–2000 mg/day; higher than standard needed in HVHF Covariates: HVHF intensity on CL
Lin, 2021 [16]	3.16 L/h (95% CI 2.83–3.40)	60.7 L (95% CI 53.2–67.5)	NR (target trough 10–20 mg/L)	16.3 ± 12.4 mg/L	36.0 ± 19.4 mg/L	Dose: No fixed regimen; Bayesian PopPK model Covariates: dopamine, TBW, burn, SCr, CRRT, age → improved trough target attainment (10–20 mg/L) to 90%
Wang, 2023 [17]	1.19 L/h (85 kg ref.; CRRT intensity covariate)	107 L	427 mg·h/L (MIC 1, efficacy threshold)	14.8 mg/L (IQR 12.3–18.2)	NR	Dose: Target AUC 427–600 mg·h/L; Effluent 20–25 → 5 mg/kg q12 h; Effluent 25–45 → 7.5 mg/kg q12 h Covariates: WT on CL; CRRT intensity on CL
Wang, 2021 [18]	1.15 L/h (pop typical)	V_c_ = 16.9 L; V_p_ = 25.9 L; Q = 7.7 L/h	400–600/400–650 mg·h/L (target range)	NR	NR	Dose: Normal alb, UFR 20–35 → 10 mg/kg q24 h; UFR 35–40 → 5 mg/kg q8 h; Low alb, UFR 20–25 → 5 mg/kg q8 h; UFR 25–40 → 10 mg/kg q12 h Covariates: alb, UFR on CL; WT on CL and V
Yamazaki, 2020 [19]	CLc: 1.35; CLp: 3.65; CLf: 1.35 ± 0.31 L/h (n = 8)	V_c_ = 59.1 L; V_p_ = 56.1 L	NR	NR	NR	Dose: LD ~27 mg/kg; MD ~10 mg/kg q24 h; severe: LD 50 mg/kg + MD 14 mg/kg q24 h; frequent TDM required Covariates: BW on Vc; SOFA score on CL
Yu, 2023 [20]	1.05 L/h (95% CI 0.72–1.53)	69.0 L	NR	NR	NR	Dose: UO ≤ 100 mL/d: 750 mg q12 h; UO > 100 mL/d: ~1000 mg q12 h (risk AUC > 600); overall: 1000 mg q12 h common; close TDM essential Covariates: 24-h urine volume on CL

alb: Albumin; AUC: Area under the curve; BW: Body weight; CI: Continuous infusion; CL: Clearance; CLc: Clearance (central); CLf: Clearance (filter); CLp: Clearance (peripheral); CRRT: Continuous renal replacement therapy; IBW: Ideal body weight; IQR: Interquartile range; LD: Loading dose; MD: Maintenance dose; MIC: Minimum inhibitory concentration; NR: Not reported; PopPK: Population pharmacokinetic; PTA: Probability of target attainment; q12 h: Every 12 h; q24 h: Every 24 h; RUO: Reduced urine output; SCr: Serum creatinine; SOFA: Sequential Organ Failure Assessment; TBW: Total body weight; TDM: Therapeutic drug monitoring; UFR: Ultrafiltration rate; UO: Urinary output; Vc: Central volume; Vd: Volume of distribution; Vp: Peripheral volume.

**Table 4:** Covariates explored versus confirmed across included PopPK studies evaluating vancomycin during CRRT.

Author, Year	Explored Covariates	Confirmed Covariates (Retained in Final Model)	Remarks
Beumier, 2013 [1]	TBW, age, effluent rate, serum alb, CRRT modality	TBW on V1; CRRT intensity on CL	Effluent rate was the strongest determinant of CL
Garreau, 2021 [2]	Ideal body weight, creatinine clearance, CRRT effluent flow	IBW on V1; effluent flow on CL	External validation confirmed effect consistency
Oda, 2020 [3]	Residual urine output, effluent rate, age, weight, alb	Residual urine output and effluent rate on CL	Bayesian model improved prediction accuracy
Udy, 2013 [5]	Age, weight, sex, effluent flow, CRRT type	NR	No covariates retained; high interindividual variability
Kirwan, 2021 [8]	Effluent rate, vasopressor use, alb, age, sex	Effluent rate and vasopressor use on CL	Vasopressor use reduced clearance
Bae, 2019 [14]	Creatinine clearance, CRRT on CL, body weight on V2	CLcr and CRRT status on CL; WT on V2	CRRT inclusion improved predictive performance
Escobar, 2014 [15]	Effluent rate, body weight, serum creatinine, age	Effluent (HVHF intensity) on CL	High-volume hemofiltration significantly increased clearance
Lin, 2021 [16]	Age, sex, TBW, serum creatinine, dopamine, burn status, CRRT use	Dopamine, TBW, burn status, SCr, CRRT, age	Composite covariate model improved target attainment
Wang, 2023 [17]	Weight, age, sex, CRRT intensity, residual diuresis	WT and CRRT intensity on CL	Confirmed effect of effluent-based clearance scaling
Wang, 2021 [18]	Alb, ultrafiltration rate, body weight, effluent rate	alb and UFR on CL; WT on CL and V	Combined effect of alb and effluent intensity
Yamazaki, 2020 [19]	Body weight, SOFA score, alb, effluent rate	BW on Vc; SOFA on CL	Filter type (PMMA) not influential
Yu, 2023 [20]	24-h urine output, weight, effluent rate	Urine output on CL	Residual diuresis significantly reduced dose requirement

Note: Explored covariates were defined as variables tested during model development but not statistically retained in the final PopPK model. Confirmed covariates were those meeting prespecified inclusion criteria (e.g., ΔOFV, *p* < 0.05) and incorporated into the final parameter equations (CL, Vd, etc.). alb: Albumin; CL: Clearance; CLcr: Creatinine clearance; CRRT: Continuous renal replacement therapy; HVHF: High-volume hemofiltration; IBW: Ideal body weight; NR: Not reported; PMMA: Polymethyl methacrylate; SCr: Serum creatinine; SOFA: Sequential Organ Failure Assessment; UFR: Ultrafiltration rate; TBW: Total body weight; Vc: Central volume of distribution; V1: Central volume; WT, weight.

**Table 5:** Methodological and validation characteristics of included population pharmacokinetic models.

Author, Year	n	Model Structure	Covariate Selection Strategy	Internal Validation	Bootstrap	External Validation
Beumier, 2013 [1]	32	2-comp (FOCE-I, NONMEM 6.1)	Biological screening + OFV-based stepwise	GOF + VPC	Yes	No
Garreau, 2021 [2]	78 (dev); 84 (val)	2-comp NLME (SAEM)	Stepwise (OFV-based)	GOF + pcVPC	No	Yes
Oda, 2020 [3]	81 patients; 199 samples	1-compartment NLME (NONMEM, FOCE-I); zero-order input (continuous infusion)	Covariate testing performed (BFR, UFR, DR, ER, weight), but none retained (no significant improvement in OFV)	GOF plots + visual diagnostics; no formal VPC reported	No	No
Udy, 2013 [5]	17 (dev); 23 (clinical Bayes-TDM evaluation); 80 concentrations	2-compartment NLME (NONMEM, FOCE-I); CLnonCRRT + CLCRRT separated	Graphical screening + forward inclusion (ΔOFV > 6.63) + backward elimination (ΔOFV > 10.83); RUO and effluent flow rate retained	GOF plots + pcVPC + CWRES diagnostics	Yes (1000 resamples; 81.9% success)	No true external dataset (sequential clinical evaluation only)
Kirwan, 2021 [8]	24 (155 conc)	1-comp NPAG (Pmetrics)	Exploratory univariable testing (−2LL/AIC/BIC-based); no covariate retained	GOF + VPC	No	No
Bae, 2019 [14]	220	2-compartment (FOCE-I, NONMEM)	Stepwise LRT-based (forward inclusion/backward elimination)	GOF + pvcVPC	Yes (1000 replicates)	No.
Escobar, 2014 [15]	9	2-comp (NONMEM 7.2; NLME)	OFV-based screening (stepwise)	GOF	Yes (1000)	No
Lin, 2021 [16]	294 (dev); 80 (external val); total 374	1-comp NLME (EM algorithm; Kinetica)	Stepwise (F-test based)	GOF plots + AIC/BIC/LL comparison	No	Yes (separate dataset + Bayesian MPE/MAE)
Wang, 2023 [17]	180 (dev); 20 (ext val)	1-comp; NLME (NONMEM)	Stepwise (OFV-based; forward inclusion/backward elimination)	GOF + NPC	Yes (1000 resamples)	Yes
Wang, 2021 [18]	11 patients (131 samples)	2-compartment; NLME (FOCEI); first-order elimination	Stepwise (OFV-based forward inclusion/backward elimination)	GOF + NPC (numerical predictive check)	Yes (2000 resamples)	No
Yamazaki, 2020 [19]	25	2-compartment NLME (first-order elimination; Phoenix NLME)	Stepwise (OFV-based; forward inclusion + backward elimination; BW → V1, SOFA → CL1)	GOF + CWRES + VPC + NPDE	Yes (1000)	No
Yu, 2023 [20]	71 patients (113 concentrations)	1-compartment NLME (FOCE-I; NONMEM)	Forward inclusion (ΔOFV > 3.84) + backward elimination (ΔOFV > 10.83); log 24-h urine volume → CL	GOF plots	Yes (1000)	No

AIC: Akaike Information Criterion; BFR, Blood Flow Rate; BIC, Bayesian Information Criterion; CL, Clearance; CLCRRT, Clearance attributable to CRRT; CLnonCRRT, Non-CRRT Clearance; CWRES, Conditional Weighted Residuals; DR, Dialysate Rate; EM, Expectation–Maximization; ER, Effluent Rate; FOCE-I, First-Order Conditional Estimation with Interaction; GOF, Goodness of Fit; LL, Log-Likelihood; LRT, Likelihood Ratio Test; MAE, Mean Absolute Error; MPE, Mean Prediction Error; NLME, Nonlinear Mixed-Effects Modeling; NPC, Numerical Predictive Check; NPAG, Nonparametric Adaptive Grid; NPDE, Normalized Prediction Distribution Errors; OFV, Objective Function Value; pcVPC, Prediction-Corrected Visual Predictive Check; RUO, Residual Urine Output; SAEM, Stochastic Approximation Expectation–Maximization; UFR, Ultrafiltration Rate; VPC, Visual Predictive Check.

## Data Availability

All data analyzed in this study are derived from published articles included in the systematic review. Extracted data are available from the corresponding author upon request.

## Supplementary Materials

The following supporting information can be downloaded at: https://www.mdpi.com/article/10.3390/pharmaceutics18030322/s1. Table S1: Detailed Search Strategy and Database Results; PRISMA 2020 checklist.
