# Supplementary material for: Optimizing Vancomycin Dosing in Continuous Renal Replacement Therapy: A Systematic Review of Population Pharmacokinetic Studies in Adult Critically Ill Patients
Source: Pharmaceutics. 2026 Mar 3;18(3):322. doi: 10.3390/pharmaceutics18030322 (PMC13029633; doi:10.3390/pharmaceutics18030322)
Supplement: Supplementary file 1 [file pharmaceutics-18-00322-s001.zip › pharmaceutics-4177244-supplementary/pharmaceutics-4177244-supplementary.pdf]

Table S1. Detailed Search Strategy and Database Results

Database searched	Platform	Years of coverage	Records	Records after duplicates removed
Medline ALL	Ovid	1946 - Present	162	162
Embase	Embase.com	1971 - Present	360	223
Web of Science Core Collection*	Web of Knowledge	1975 - Present	261	92
CINAHL Plus	EBSCO	1982 - Present	36	3
**Total**			**819**	**480**

*Science Citation Index Expanded (1975-present) ; Social Sciences Citation Index (1975-present) ; Arts & Humanities Citation Index (1975-present) ; Conference Proceedings Citation Index- Science (1990-present) ; Conference Proceedings Citation Index- Social Science & Humanities (1990-present) ; Emerging Sources Citation Index (2005-present)

No other database limits were used than those specified in the search strategies

Medline 162

(Pharmacokinetics / OR Vancomycin /pk OR (pharmacokinetic* OR ((absorption* OR accumulation* OR activation* OR adsorption* OR bioavailability* OR clearance* OR dialysability* OR diffusion* OR disposition* OR distribution* OR elimination* OR excretion* OR half-life* OR inactivation* OR metabolism* OR penetration* OR release* OR removal* OR retention*) ADJ3 (drug* OR vancomycin* OR minim*-inhibit*-concentrat* OR concentration-time*))).ab,ti,kw. OR (absorption* OR accumulation* OR activation* OR adsorption* OR bioavailability* OR clearance* OR dialysability* OR diffusion* OR disposition* OR distribution* OR elimination* OR excretion* OR half-life* OR inactivation* OR metabolism* OR penetration* OR release* OR removal* OR retention* OR minim*-inhibit*-concentrat* OR concentration-time* OR pk).ti.) AND (Vancomycin / OR (vancomycin*).ab,ti,kw.) AND (Continuous Renal Replacement Therapy/ OR * Renal Replacement Therapy/ OR (((contin* OR high-volum*) ADJ3 (renal-replacement* OR kidney*-replacement* OR hemofiltrat* OR hemodialy* OR hemodiafiltr* OR haemofiltrat* OR haemodialy* OR haemodiafiltr* OR ultrafiltrate*)) OR CVVH OR CVVHD OR CVVHDF).ab,ti,kw. OR (renal-replacement* OR kidney*-replacement* OR hemofiltrat* OR hemodialy* OR hemodiafiltr* OR haemofiltrat* OR haemodialy* OR haemodiafiltr* OR ultrafiltrate*).ti.) NOT (exp animals/ NOT humans/) AND english.la. NOT (In Vitro Techniques / OR Case Reports / OR (in-vitro* OR case-report*).ti.) NOT ((exp child OR exp infant/ OR adolescent) NOT exp adults/)

Embase 360

(pharmacokinetics/exp OR 'pharmacokinetic parameters'/exp OR 'minimum inhibitory concentration'/de OR (pharmacokinetic* OR ((absorption* OR accumulation* OR activation* OR adsorption* OR bioavailability* OR clearance* OR dialysability* OR diffusion* OR disposition* OR distribution* OR elimination* OR excretion* OR half-life* OR inactivation* OR metabolism* OR penetration* OR release* OR removal* OR retention*) NEAR/3 (drug* OR vancomycin* OR minim*-inhibit*-concentrat* OR concentration-time*))):ab,ti,kw OR (absorption* OR accumulation* OR activation* OR adsorption* OR bioavailability* OR clearance* OR dialysability* OR diffusion* OR disposition* OR distribution* OR elimination* OR excretion* OR half-life* OR inactivation* OR metabolism* OR penetration* OR release* OR removal* OR retention* OR minim*-inhibit*-concentrat* OR concentration-time* OR pk):ti) AND (vancomycin/exp OR (vancomycin*):ab,ti,kw)

AND ('continuous renal replacement therapy'/exp OR 'renal replacement therapy'/mj OR (((contin* OR high-volum*) NEAR/3 (renal-replacement* OR kidney*-replacement* OR hemofiltrat* OR hemodialy* OR hemodiafiltr* OR haemofiltrat* OR haemodialy* OR haemodiafiltr* OR ultrafiltrate*)) OR CVVH OR CVVHD OR CVVHDF):ab,ti,kw OR (renal-replacement* OR kidney*-replacement* OR hemofiltrat* OR hemodialy* OR hemodiafiltr* OR haemofiltrat* OR haemodialy* OR haemodiafiltr* OR ultrafiltrate*):ti) NOT [conference abstract]/lim NOT ([animals]/lim NOT [humans]/lim) AND [english]/lim NOT ('in vitro study'/exp OR 'case report'/de OR (in-vitro* OR case-report*):ti) NOT (juvenile/exp NOT adults/exp)

Web of science 261

(TS=(pharmacokinetic* OR ((absorption* OR accumulation* OR activation* OR adsorption* OR bioavailability* OR clearance* OR dialysability* OR diffusion* OR disposition* OR distribution* OR elimination* OR excretion* OR half-life* OR inactivation* OR metabolism* OR penetration* OR release* OR removal* OR retention*) NEAR/2 (drug* OR vancomycin* OR minim*-inhibit*-concentrat* OR concentration-time*))) OR TI=(absorption* OR accumulation* OR activation* OR adsorption* OR bioavailability* OR clearance* OR dialysability* OR diffusion* OR disposition* OR distribution* OR elimination* OR excretion* OR half-life* OR inactivation* OR metabolism* OR penetration* OR release* OR removal* OR retention* OR minim*-inhibit*-concentrat* OR concentration-time* OR pk)) AND TS=((vancomycin*)) AND (TS=(((contin* OR high-volum*) NEAR/2 (renal-replacement* OR kidney*-replacement* OR hemofiltrat* OR hemodialy* OR hemodiafiltr* OR haemofiltrat* OR haemodialy* OR haemodiafiltr* OR ultrafiltrate*)) OR CVVH OR CVVHD OR CVVHDF) OR TI=(renal-replacement* OR kidney*-replacement* OR hemofiltrat* OR hemodialy* OR hemodiafiltr* OR haemofiltrat* OR haemodialy* OR haemodiafiltr* OR ultrafiltrate*)) NOT TI=((in-vitro* OR case-report*)) NOT TS=((juvenile* OR child* OR infan* OR adolescen* OR pediatr* OR paediatr*) NOT adult*) NOT DT=(Meeting Abstract OR Meeting Summary) AND LA=(english)

CINAHL 36

(MH Pharmacokinetics OR TI(pharmacokinetic* OR ((absorption* OR accumulation* OR activation* OR adsorption* OR bioavailability* OR clearance* OR dialysability* OR diffusion* OR disposition* OR distribution* OR elimination* OR excretion* OR half-life* OR inactivation* OR metabolism* OR penetration* OR release* OR removal* OR retention*) N2 (drug* OR vancomycin* OR minim*-inhibit*-concentrat* OR concentration-time*))) OR AB(pharmacokinetic* OR ((absorption* OR accumulation* OR activation* OR adsorption* OR bioavailability* OR clearance* OR dialysability* OR diffusion* OR disposition* OR distribution* OR elimination* OR excretion* OR half-life* OR inactivation* OR metabolism* OR penetration* OR release* OR removal* OR retention*) N2 (drug* OR vancomycin* OR minim*-inhibit*-concentrat* OR concentration-time*))) OR TI(absorption* OR accumulation* OR activation* OR adsorption* OR bioavailability* OR clearance* OR dialysability* OR diffusion* OR disposition* OR distribution* OR elimination* OR excretion* OR half-life* OR inactivation* OR metabolism* OR penetration* OR release* OR removal* OR retention* OR minim*-inhibit*-concentrat* OR concentration-time* OR pk)) AND (MH Vancomycin OR TI(vancomycin*) OR AB(vancomycin*)) AND (MH Continuous Renal Replacement Therapy OR MM Renal Replacement Therapy OR TI(((contin* OR high-volum*) N2 (renal-replacement* OR kidney*-replacement* OR hemofiltrat* OR hemodialy* OR hemodiafiltr* OR haemofiltrat* OR haemodialy* OR haemodiafiltr* OR ultrafiltrate*)) OR CVVH OR CVVHD OR CVVHDF) OR AB(((contin* OR high-volum*) N2 (renal-replacement* OR kidney*-replacement* OR hemofiltrat* OR hemodialy* OR hemodiafiltr* OR haemofiltrat* OR haemodialy* OR haemodiafiltr* OR ultrafiltrate*)) OR CVVH OR CVVHD OR CVVHDF) OR TI(renal-replacement* OR kidney*-replacement* OR hemofiltrat* OR hemodialy* OR hemodiafiltr* OR haemofiltrat* OR haemodialy* OR haemodiafiltr* OR ultrafiltrate*)) NOT (MH

animals+ NOT MH humans+) AND LA(English) NOT (MM In Vitro Studies OR MM Case Studies OR TI(in-vitro* OR case-report*)) NOT ((MH child OR MH infant+ OR MH adolescent) NOT MH adults+)
